# Large Posterior Communicating Artery Aneurysm: Initial Presentation with Reproducible Facial Pain Without Cranial Nerve Deficit

**DOI:** 10.5811/westjem.2016.8.30457

**Published:** 2016-09-29

**Authors:** Stacie Zelman, Michael C. Goebel, David E. Manthey, Seth Hawkins

**Affiliations:** Wake Forest University Baptist Medical Center, Department of Emergency Medicine, Winston-Salem, North Carolina

## Abstract

Unruptured posterior communicating artery (PCOM) aneurysms can be difficult to diagnose and, when large (≥ 7mm), represent a substantial risk to the patient. While most unruptured PCOM aneurysms are asymptomatic, when symptoms do occur, clinical manifestations typically include severe headache (HA), visual acuity loss, and cranial nerve deficit. This case report describes an atypical initial presentation of a large unruptured PCOM aneurysm with symptoms mimicking trigeminal neuralgia, without other associated cranial nerve palsies or neurologic deficits. The patient returned to the emergency department four days later with a HA, trigeminal neuralgia, and a new cranial nerve III palsy. After appropriate imaging, she was found to have a large PCOM aneurysm, which was treated with surgical clipping with significant improvement in patient’s symptoms.

## INTRODUCTION

Intracranial aneurysms are estimated to have a prevalence of 3.2% in the United States. Patients have a mean age at diagnosis of 50 years.^1^ Unruptured aneurysms are often asymptomatic and may be discovered as incidental findings. When intracranial aneurysms rupture they result in subarachnoid hemorrhage (SAH) and/or subdural hemorrhage (SDH), with fatality rates of 40%. Of patients who survive rupture of an intracranial aneurysm, 66% suffer from permanent symptoms, most with neurological deficits.^2^,^3^ Recent evidence has suggested posterior communicating artery (PCOM) aneurysms may have a higher rate of rupture than anterior circulation aneurysms, with a five-year risk of rupture of 14.5% for aneurysms 7–12mm. Thus, identifying these aneurysms before they rupture is key to improving patient outcomes.^4^ Symptoms suggestive of PCOM aneurysms vary, but most sources agree that the presence of ocular motor nerve palsy and severe SAH-like headache are the most common.^5^,^6^

We present the case of a patient who initially presented to the emergency department (ED) with unilateral, reproducible facial pain consistent with trigeminal neuralgia (with migraine in the differential) and was treated as such with some relief of her symptoms. It was not until her second visit to the ED that she exhibited the neurological deficit of cranial nerve III (CN III) commonly associated with large PCOM aneurysm.

## CASE REPORT

A 42-year-old woman presented to the ED with two weeks of right-sided headaches similar to her previous headaches. Past medical history was remarkable for headaches that usually resolved spontaneously, but this one had not. In addition, she also noted right-sided facial pain and sensitivity. The facial pain was reproducible and originated behind her right ear, with radiation across the face. The patient denied any facial droop or weakness. She also denied any changes in visual acuity, but did complain of a foreign body sensation in her eye.

The patient’s initial vital signs were a blood pressure of 144/79, pulse 72, respirations 17, temperature 99.7°F, and SpO2 100% (room air). On physical exam, the patient exhibited no physical distress. Her neurologic exam was unremarkable with cranial nerves, strength, and gait tested and noted to be intact. An ocular exam, including intraocular pressure testing and fluorescein evaluation, was also noted to be negative for pathology. She was treated with metoclopramide and ketorolac for her migraine while in the ED, with some relief of symptoms. Because her symptoms also appeared consistent with trigeminal neuralgia, she was discharged with a trial of carbamazepine.

The patient returned to the ED four days later with a chief complaint of right eye pain and pressure with associated blurred vision. The patient’s sister also noted that the patient’s eyelid appeared “droopy.” The patient also complained of some numbness to her right side. Physical exam was remarkable for a new marked ptosis of the right eyelid and miosis of the right pupil. The rest of the physical exam was unremarkable. Based on these new physical findings, an emergent computed tomography angiography (CTA) of the head and neck was performed. The imaging revealed 7mm by 4mm bilobed posterior directed PCOM saccular aneurysm, which is demonstrated in the figure.

Once imaging was completed, an emergent neurosurgery consult was obtained. The patient was maintained on strict blood pressure control (systolic blood pressure less than 140), and transferred to the neurosurgery intensive care unit until the she could be taken to the operating room. In the operating room a right pterional craniotomy was performed followed by clipping of the right posterior communicating artery. The patient also received dexamethasone on the day of the surgery and post-operative day one.

The patient progressed remarkably well after her surgical procedure. She had immediate relief of her facial pain and significant improvement of her CN III palsy and was discharged home three days post-operation. At her outpatient follow-up appointment two weeks post-operation she reported complete resolution of her headaches and had completely normal extra-ocular movements and only mild ptosis on exam.

## DISCUSSION

This case helps illustrate why patients presenting with a headache and cranial nerve irritation may require advanced imaging for a mass. The differential of facial pain is broad and contains treatable conditions with high morbidity and/or mortality such as intracranial aneurysms, masses, bleeds or acute angle closure glaucoma, in addition to the more benign diagnoses of primary headache or trigeminal neuralgia. While facial pain without neurological deficits is a rare presentation of a large PCOM aneurysm, full neurologic and ocular exams should be considered in patients presenting with facial pain, with the possibility of neuroimaging based on clinical findings. As seen in this case, such patients can present with associated headache as well as prior history of headaches. With headaches being a common chief complaint, comprising 4.5% of all ED visits in U.S., and the overwhelming majority of them being benign primary headaches, vigilance for the possibility of more serious secondary headaches is difficult to maintain but important.^7^

All patients presenting to the ED with facial pain, especially if it is new in onset or different from previous episodes, should have a thorough physical exam including complete neurological and ophthalmological exam, with specific focus on cranial nerve deficits and intraocular pressures. Any abnormalities on this exam should prompt the provider to strongly consider further investigation with neuroimaging, as they are potentially caused by a life-threatening intracranial process such as a large PCOM aneurysm. In this case, a detailed neurologic exam was performed, but irritation of the trigeminal nerve was attributed to a peripheral cause instead of a central cause. In this patient’s case, it appears that her trigeminal neuralgia was caused by a central irritation due to the compression by the bilobed aneurysm. This is likely why her pain (and CN III deficit) resolved after surgery. If neuroimaging is not pursued during the patient’s ED visit (as it was not in this patient’s first visit due to lack of perceived central neurological deficits), it is imperative that strict ED return precautions be given. These should include neurological deficits such as vision or eye movement deterioration as well as worsening of the patient’s pain. As in this case, such return precautions can lead to timely reevaluation of the patient, where changes in physical exam can be identified and further workup performed.

Upon identification of large (≥ 7mm) or clinically symptomatic intracranial aneurysms, emergent neurosurgical consultation is indicated, as interventions such as surgical clipping are associated with improved clinical outcomes and cost effectiveness.^8^

This case illustrates the importance of considering posterior circulation aneurysms in patients with new headaches or changing symptoms specifically involving new pain of the face or eyes. Thorough neurological and ophthalmological examination including cranial nerve function and bilateral IOPs should be performed on these patients when they present to the ED. Good follow-up instructions and return precautions, including development of cranial nerve palsies, is important so the patient knows what symptoms to monitor for and when to return for reevaluation. Early surgical clipping of large and/or symptomatic aneurysms can improve patient quality of life and mortality.

## Figures and Tables

**Figure:**
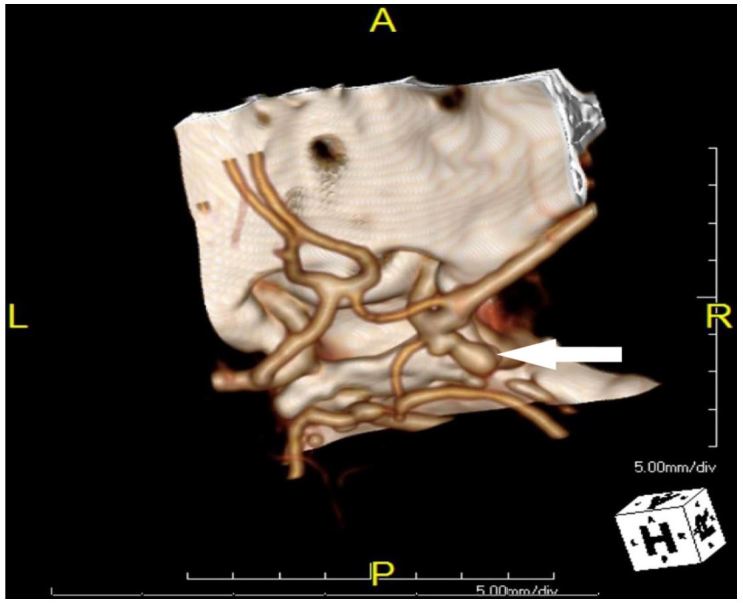
Three-dimensional contrast enhanced computed tomography reconstruction showing bilobed right posterior communicating artery aneurysm.
